# The Individual and the Collective: Sociological Influences on Lacan's Concept of the Relation Subject—Other

**DOI:** 10.3389/fpsyg.2018.00614

**Published:** 2018-05-15

**Authors:** David Schrans

**Affiliations:** Department of Psychoanalysis and Clinical Consulting, Faculty of Psychology and Educational Sciences, Ghent University, Ghent, Belgium

**Keywords:** Lacan, individual, collective, Durkheim, Mauss, Lévi-Strauss, subjective assertion, family complexes

## Abstract

In this conceptual analysis we discuss the sociological influences on Lacan's conceptualization of the relation between the subject and the other. In his writings predating World War II Lacan defines this relation in terms of identification. However, from 1953 onward he defines it in terms of the subject of speech and the Symbolic Other. It is a popular notion to characterize this change in terms of a radical breach, influenced by the reading of Lévi-Strauss. However, through a close reading of both Lacan's early writings and their Durkheimian influences we will demonstrate that what has changed, is Lacan's conceptualization of the relation between the individual and the collective. This change was gradual rather than sudden. Moreover, it can be situated within the theoretical evolution of the contiguous fields of sociology, anthropology and psychoanalysis. Thus we reject the idea of a breach within his own thought and with what came before him. We will establish our point through a summary of how the relation between the individual and the collective was theorized before Lacan. Durkheim conceptualized this relation as dual: the individual and the collective are radically separated. Mauss attempted to unify the field of anthropology through the holistic concept of the total man. In Lévi-Straus's formalization the individual becomes a function within a structured, Symbolic system. Finally, a reading of Lacan's publications concerning the notion of the logic of the collective will testify to his attempts at formulating a notion of the subject that asserts itself against this collective while at the same time retaining its nature of a logical function. This is the conundrum that Lacan will confront time and again throughout his teachings. Lévi-Strauss merely provided him with the methodological tools of structural anthropology that helped him refine the interrogations that he had already begun.

## Introduction

A popular notion among Lacanian scholars is the occurrence of a radical shift from a Lacan preoccupied with the Imaginary order to a Lacan focused on the Symbolic. Where the former is centered around his theory on the mirror stage (Lacan, 2006 [1949]), the latter has come about through his reading of the anthropologist Claude Lévi-Strauss (Zafiropoulos, 2001; Zafiropulos, 2010 [2003]) and is inaugurated by his presentation entitled *The function and field of speech and language in psychoanalysis* in 1953 (Lacan, 2006 [1953b]).

In this paper we reject the notion of a radical shift from the Imaginary to the Symbolic. What is more, we defend the idea that Lacan's evolving conceptual framework can be situated within a broader intellectual history of anthropological and sociological thought concerning the relation between the individual and the collective. Hence, we neither accept a breach within his own thought, nor with what came before him.

The relation between the individual and the collective has occupied psychoanalysis since Freud's writings on group psychology (Freud, 1955 [1921]) and has been carried on by psychoanalysts such as Bion and Rickman, both of whom have influenced Lacan (2001 [1947]). However, it has also been one of the tenets of sociology and anthropology. The notion of individuality and the influence of the collective is central in Durkheim's work, which ended in an impasse where both were radically separated. Marcel Mauss attempted to resolve this impasse with his notion of the total man and Lévi-Strauss formalized this notion and its implications. In this paper we will focus on the period between Lacan's writings on the family, where this relation is based on identification and heavily influenced by Durkheim, and his writings on the Symbolic, where it is based on language and heavily influenced by Mauss and Lévi Strauss.

## Durkheim and lacan on the family

As both Freud (1955 [1921]) and Lacan (2001 [1938]) noted, the foundations for man's social life, and thus his relation to the collective, are laid down in the family. Zafiropoulos (2001) states that Lacan was influenced by Durkheim in his writings on the family. We will therefore subject Durkheim's teachings on the family to a close reading and compare it with Lacan's paper on the family complexes. Finally, we will confront our reading with Zafiropoulos' interpretation, which states that Lacan eventually diverted from Durkheim through a new interpretation of the notion of the father inspired by Lévi-Strauss.

### Durkheim on the contraction of the family

Durkheim stated that the family is a social institution subject to a cultural evolution, with the conjugal family as its final conclusion. It is not merely a biological affair, but has moral and judicial implications which are protected by the collective in which the family is embedded. The evolution of the institution of the family is determined by what Durkheim (1975 [1892]) calls the *law of contraction*: throughout history the number of relatives which constitute a family has progressively diminished. The historical starting point of the family is the clan. In this social structure a totem or alleged forefather is responsible for the creation of society and forms the center of family life. Members of a clan were both relatives and fellow citizens (Lamanna, 2002). In these societies there was no notion of an individual and the different members of the group were hardly distinguished from one another. Only when the clan ceased to be nomadic and started to attach value to the territory on which it lived, did family and clan become two separate entities. A broad, amorphous family system became distinguished from a political and territorial clan organization (Durkheim, 1984 [1893]). Families with a patrilineal or matrilineal structure originated within the clan (Durkheim, 1975 [1892]). A further contraction was realized by the agnatic families, which were smaller and more egalitarian than the totem based families. These families were centered around shared possessions, rather than religion and could be either patrilineal or matrilineal.

This differs from the Roman, patriarchal families, which were strictly patrilineal and governed by the principle of *patria potestas*. The father represented the group and his authority over its members and possessions was absolute. This contrasts with the German paternal family where paternal dominance was less strict. The son could emancipate himself and leave the family on his own accord.

The conjugal family is the family structure discerned by Durkheim in French society of his day. It is a further contraction of the paternal rather than of the patriarchal family as the latter was too strict to allow for any further contraction. The only permanent elements within this system are husband and wife, although secondary zones of kinship (i.e., relatives up to the sixth degree), as remnants of the previous structures, still existed. A child is dependent on its father until it is married. As Zafiropoulos (2001) correctly points out, with the contraction of the family the disciplinary rights of the father have greatly diminished. However, the interference of the state has increased. In France as of 1889 the father can even be set out of this paternal rights by the state. According to Durkheim, state intervention was a necessary prerequisite for the existence of the conjugal family. Whereas kinship relations in societies constituted by patriarchal families could only be broken off under the authority of the father, in the case of those based on the conjugal family the state must provide its approval in cases of divorce or adoption. Durkheim warns us however that with the contraction of the family, individuality and the pursuit of purely individual goals have increased. When the individual “looks for his purpose within himself, he falls into a state of moral misery which leads him to suicide” (Durkheim, 1975 [1892], p. 236). Yet, according to Lamanna (2002) Durkheim is not necessarily pessimistic where it concerns the increase of individuality and the decrease of paternal authority. The former gave rise to individual freedom and the latter consolidated the ties between the members of society through state intervention.

### Lacan on the family complexes

According to Zafiropoulos (2001) Lacan was heavily influenced by Durkheim's writings on the family when he wrote his own contribution on the family complexes (Lacan, 2001 [1938]). Lacan states that the process of subjective development is structured by three fundamental complexes which center around three imagoes: the weaning complex with the imago of the mother, the complex of intrusion with the imago of the rival and the Oedipus complex with the imago of the father. The structuring of these complexes takes place within the family as a historically determined institution. However, in our modern, Western families (for which Lacan uses the term “conjugal families”) the father has become progressively absent due to the “contraction of the institution of the family” (Lacan, 2001 [1938], p. 27). As a consequence the Oedipus complex in Western society has started to falter, which explains the burgeoning of modern forms of psychopathology such as the character neuroses.

The first complex the human infant encounters, the weaning complex, is “the most primitive complex in psychic development” and “forms the basis of the most archaic and stable sentiments uniting the individual and the family” (Lacan, 2001 [1938], p. 30, trans. Cormac Gallagher). This complex originates from the separation of the infant from the womb, which, for man, is always a premature separation. The repercussion of its prematurity is the universally shared call of the young human for the social group, and in the first place the family, which is in fact a call for some sort of social function which meets the needs emanating from this vital insufficiency of the first years. The first form this social connection with the outside world takes on is the imago of the mother as a sublimation of the mother, a bringing into form and recognizing her as an answer to the vital insufficiency. It is the first connection to the other the infant makes. It is also the connection upon which all the consecutive connections with the other will be made. A successful transition of the weaning complex is therefore paramount to social development.

As important as the imago of the mother is, it is marked by a strange ambivalence. The longing for the maternal imago can become a longing for the state before birth, and as such instigate the death drive. Because of this ambivalence the lure of death, of a return to the tranquil, inanimate state of life before birth, remains present in the ambiguous form of the imago of the mother. The complex of intrusion offers a solution to these summons of death through a confrontation with the double, archetypally represented in the form of the brother. This complex roused by the first realization of the presence of a sibling, and of the feelings of jealousy concerning the mother this provokes. Lacan cautions us that we must not confuse human jealousy with biological rivalry. For at its most fundamental, jealousy presupposes mental identification. Lacan considers identification as primary, the aggressiveness it provokes as secondary. Lacan's description of the complex of intrusion is an early form of his theory on the mirror stage (Lacan, 2006 [1949]). He states that through the recognition of its own image in the mirror the human infant regains the unity it once experienced *in utero*.

At the same time the body is experienced as unity, the world, which was equally perceived as fragmented, is organized by reflecting the forms of the body. Consequently, Lacan declares the mirror image to be a good symbol for the reality as it is experienced at that moment in human development. The world in this phase is a narcissistic world in which the image of the double is central and there is no place for others (as truly “other”). Indeed, the experience of the other as a mirror image does not help the subject to break through the affective isolation caused by its prematurity. However, as the formation of the ego through identification with an external image occurs, the drama of jealousy and sibling rivalry is being played out. Identification with the unweaned sibling provokes aggressiveness because it triggers the maternal imago and thus the desire for death. This is why Lacan states that aggressiveness is secondary to identification: the identification with the other awakens the primary masochism (i.e., the death drive) of the weaning complex. However, this primary masochism can be overcome if it is transformed into sadism in the form of rivalry. Through identification the infant can fix one of the poles of primary masochism and turn it into aggressiveness toward the unweaned sibling. “Thus the non-violence of the primordial form of suicide engenders the violence of the imaginary murder of the sibling” (Lacan, 2001 [1938], p. 40, trans. Cormac Gallagher). Consequently the other is recognized as truly *other*. This is why Lacan states that jealousy is the archetype of all social sentiments. However, the complex of intrusion can also end in an imaginary impasse where the ego and the alter-ego are not distinguished. This can lead to serious forms of psychopathology (e.g., paranoia).

Finally, the Oedipus complex installs two fundamental, psychological instances: repression and the Ego-ideal. Contrary to Freudian doctrine, which recognizes the father as the primary agent of castration, Lacan states that the original cause for repression stems from the lure of death present in the imago of the mother. The sexual desires of the Oedipus complex re-activate the desire for the mother and thus the ancient death drive which is thereupon repressed. The father, as the one who opposes this desire for the mother, figures only as a secondary source for repression. As such, repression paves the way for yet another form of identification with the rival, but this time as an Ego-ideal: “the ideal of virility in the boy, and in the girl the ideal of virginity” (Lacan, 2001 [1938], p. 56, trans. Cormac Galagher). However, Lacan remarks that not every society accords the same place to the father and its successful development depends largely on the extent to which both the repressive and the sublimatory functions are united in the imago of the father. In the Trobriand of Melanesia, for example, the repressive function is attributed to the maternal uncle, the sublimatory function to the biological father. The effect is a relative absence of neurosis and a great rigidity on the level of cultural productions. In our Western society “the father […] is always lacking in some way or another, whether he be absent or humiliated, divided or a sham” (Lacan, 2001 [1938], p. 61, trans. Cormac Galagher) and the reason for this Lacan finds in “the same social progress that makes the family evolve toward the conjugal form [and] increasingly submits it to individual variations” (Lacan, 2001 [1938], p. 74, trans. Cormac Galagher). When this happens the dialectic of sublimation is negated and libidinal energy exhausted, which eventually leads to character neurosis.

Zafiropoulos (2001) states that Lacan's views betray a Durkheimian influence because he links the degradation of the Oedipus complex to the contraction of the family. The author claims that Lacan is strictly non-Freudian as long as he is influenced by Durkheim. In *Totem and Taboo* Freud (1953 [1912]) stated that human society commenced with the murder of the primal father. As such, the degraded, dead father is at the foundation of every human society and not a historical contingency. Only with the discovery of structural anthropology through the writings of Lévi-Strauss would Lacan be able to recognize the structurally lacking father function and commence his return to Freud. Although we acknowledge the presence of Durkheimian influences in Lacan's text from 1938, we find Zafiropoulos' arguments on his transition from Durkheimian/non-Freudian to structuralist/Freudian to leave a lot to be desired. First, although he cites Lévi-Strauss' work as being responsible for this sudden change in Lacan's thinking Zafiropoulos never actually demonstrates how this work led to an interpretation of the father as structurally lacking. When discussing this change, his main reference is French writer Paul Claudel and his play *Le père humilié*. Second, we have seen that with the contraction of the family there has been a shift in authority from the father to the state. Even if Durkheim writes about the degradation of the father, he takes into account other forms of authority and law. When discussing Durkheim's views on the relation between the individual and the collective, we will see that this shift can be explained by an evolution in this relation. And last but certainly not least, we have difficulty accepting that such a slow and arduous process as the evolution of one's thinking, Lacan's in this case, is marked by sudden revolutions. During an analysis something can befall the patient and create a new insight—but every analyst knows that revolutions are very rare in the consulting room. There is always the process of working through to take into account. Therefore, it is more interesting to look at the historical development that links Durkheim to Lacan *through* Lévi-Strauss, because the latter wouldn't have been possible without Durkheim. In what follows, we will present the reader with the evolution of the conceptualization of the relation between the subject and the Other (in sociological terms: between the individual and the collective) from Durkheim to Lacan. This point of view will better allow us to link the gradual evolution of Lacan's thinking to the developments in sociology and anthropology that preceded him.

## Durkheim and the opposition between individual and collective

In *The Division of Labor in Society* Durkheim (1984 [1893]) describes two forms of relation between the individual and the collective: mechanical solidarity, a relation based on similarity, and organic solidarity, a relation based on difference. Between these two forms he describes an evolution. Primitive societies are primarily based on mechanical solidarity. The members of these societies are hardly differentiated. This form of solidarity is represented in “the totality of beliefs and sentiments common to the average members of a society” which then “forms a determinate system with a life of its own” (Durkheim, 1984 [1893], p. 39). This system is the collective or common consciousness, which cannot be located within a single physical substratum but is present in its entirety in every member of society. Nevertheless, it exists independent from these individuals: “Individuals pass on, but it abides” (Durkheim, 1984 [1893]). It does not change from one generation to the other, but links the different generations to each other. The origin of the collective unconscious lies in the confrontation of shared feelings and representations. Central to Durkheim's theory on the collective consciousness is the notion of vitality. Consciousness, whether it be individual or collective, derives its force, its vitality, from strong representations. The confrontation of shared representations within a society gives these collective representations a greater vitality, which largely surpasses the vitality of individual representations. This way of conceptualizing consciousness has several consequences. First, Durkheim concludes a split within the human mind between an individual and a collective consciousness where the latter appears as “an echo resounding within ourselves of a force that is alien, one moreover superior to that which we are ourselves” (Durkheim, 1984 [1893], p. 56). Second, because of its greater vitality the collective consciousness appears as a moral force. It is also the strongest form of authority. When a certain representation or act goes against these collective representations, against the moral order and greatest authority, this provokes a heavy emotional response from the group. Therefore, Durkheim states that penal law is the most common expression of the collective consciousness. Last, it also implies that this authority is not a social function, which receives a relative importance according to the society in which it occurs, but *represents the society as a whole*. For example, in the Roman, patriarchal families the father did not incarnate a specific social function but represented the group and its moral ascendancy as such.

Whereas mechanical solidarity is based on similarity, organic solidarity is based on difference and specialization. More specifically, it is based on what Durkheim calls the “division of labor.” As a society grows larger and more complex the functions the different members fulfill become more diversified. This in turn increases the degree of individuality within that society. This radically changes the relation between the members because now they all depend upon one another (like the organs that constitute a body), whereas in societies based on mechanical solidarity there was virtually no differentiation and every member could easily be replaced by any other. According to Durkheim the standard expression of this form of solidarity is not penal law, but contractual law. The relation between the members of these societies are consolidated through contract. However, this does not mean that such societies are merely based on free exchange where relations are fleeting and exist only for the duration of the contract. The state regulates and harmonizes the different (professional, domestic, etc.) relations between the individuals by offering a legal framework within which contracts can be made. Moreover, the state is as dependent on the different members of society as they are on it and on each other. Society no longer treats its members as things over which it has rights, but as cooperating members on which it depends and with regards to whom it has certain obligations. Thus Durkheim explains the degraded authority of the father, on which Lacan based his theories on the Oedipus complex in 1938, through a change in the nature of the relation between the individual and the collective. In the patriarchal societies of yore, members were hardly differentiated and the father represented the group as a superhuman authority. In modern societies based on the division of labor this authority shifts to the state as a governing instance which is as dependent on the individuals as they are on the collective. That is why Durkheim states that “each time that we find ourselves faced with a mechanism of government endowed with great authority we must seek the reason not in the particular situation of those governing, but in the nature of the societies that they govern” (Durkheim, 1984 [1893], p. 143–144).

Even though he discerns an evolution toward individuality, Durkheim continues to stress the importance of the collective representations and their effect on the individual. In a paper written with his nephew, Marcel Mauss (Durkheim and Mauss, 1903), he describes the impact of social morphology (i.e., the composition and structure of a society) on the classification systems used by the members of a given society. Whether it is a primitive classification of plants based on an animistic belief, or a scientific classification based on genetics, man categorizes the world that surrounds him in representational systems that have a certain structure and hierarchy. Durkheim states that these systems of classification are shaped by a process influenced by all sorts of external elements, the most fundamental of which is the social organization of a society. Therefore, many of the primitive classification systems reflect the morphology of the society from which they originate. However, once these representational systems have gained their independence from the social structures from whence they sprung, they function according to their own logic.

In conclusion we can state that Durkheim's collective consciousness contains its own collective representations, has its own vitality and functions according to its own laws. What is more, Durkheim (1924 [1898]) maintains a strict separation between the collective and the individual. The collective conscious exists both as an independent entity and resides *in its entirety* in every individual, albeit in an unconscious form. He deduces this from the fact that the collective representations appear to come from outside the individual and impose themselves in a coercive manner, under the form of moral obligations.

Karsenti (1997) states that Durkheim's conceptualization brings sociology in an awkward position. He has materialized the collective (*chosification*), made it into a collective consciousness that is unconscious on an individual level. As such, sociology can only study the social in the light of this irreducible dualism, where the collective has a certain ascendancy over the individual. This is due to the fact that for Durkheim a representation can only exist if it represents something *to somebody*. In order to allow for the idea of an unconscious representation he has to dissociate the notion of representation and individual consciousness. Subsequently, he postulates the existence of a collective consciousness that exists alongside the individual consciousness, because only then the collective representations can be unconscious *for us individuals* but not *as such*. It is only afterwards that these collective representations appear, not as the product of some impersonal subject, but as the effect of their insertion on the level of the limited, individual consciousness. Thus the notion of representation lead Durkheim into an impasse where the individual and the collective are strictly dissociated.

## Mauss and the total man

Mauss will furnish sociology with a new object: the total man (*l'homme total*). In a shift from sociology to anthropology, which studies man in all his aspects (social, individual, biological, etc.), he will rectify Durkheim's decentering of the individual and reunite the individual and the collective. The social forms but a single aspect of this total man. Maus's is no longer a dualistic approach, but one that focuses on the complex and dialectical relation between the individual and the collective in an effort to expose its underlying rules and structures, rather than its representations. Mauss defends the notion of a gradual difference between individual and collective. Individual representations can permeate the collective and vice versa. Every individual possesses a fragment of the totality of collective representations, which are moreover marked by the singularity of the individual.

He finds one of the most convincing examples of these transitions between collective and individual in the physical and psychological effects on the individual of the collective suggestion of death. Mauss (1950 [1926]) describes how in certain primitive cultures in Australia and New-Zealand individuals die because of magical or moral causes suggested by collectively shared beliefs. The individual dies not because of some physical disorder or any personal conviction or deed, but because of the way in which the collective manifests itself on an individual level—or, which comes down to the same in Mauss' view, the way in which the individual positions itself within the collective. Subjective consciousness and the will to live pale in comparison to the collective necessity to die. However, this is not, as it would be in a Durkheimian logic, because the collective representations act on the individual as an external cause, but because the individual assumes a given position within the social structure of the tribe. Hence, this state often coincides with a breach of community. Through magic or the committing of a crime the individual breaks off with those forces which had up until then supported it. For example, the Wakelbura who eats prohibited game will become sick, consume himself and eventually die while gasping out the sounds of the animal in question^1^.

According to Mauss the destructive effect of this collective suggestion takes place on the joint between man's social and biological nature, albeit minimally mediated by his individual consciousness. Karsenti (1997) states that this is only possible if one supposes that the collective resides *within* the individual, alongside the individual will to live. Mauss explains this phenomenon with the assumption that psychological, physical and social forces coincide in the individual, and rejects the idea of a strict dissociation between the collective and the individual. As such, this phenomenon is structured along these three axes: (1) an individually experienced conviction to die responds to (2) a collectively shared suggestion to die, upon which (3) a physical event follows.

Contrary to Durkheim, who took the collective representations to be the primary object of sociology, Mauss wants to study the total individual through anthropology. He defends a return to man in his most concrete form. This is a psychological and biological individual who appropriates a fragment of the collective in a process called socialization. Man is totally individual and totally collective. Moreover, both orders are subject to the same logic and the same laws. This totality can equally be found in language. Sociology and anthropology would benefit from adapting the methods of linguistics, because they form a science that studies a phenomenon that is both physiological, psychological and social. Lévi-Strauss will be the one who takes up the challenge and applies the methods of linguistics to social structures.

## Lévi-strauss and the symbolic function

For our discussion of Lévi-Strauss' elaboration of Mauss' symbolic framework, we will rely heavily of his *Introduction to the work of Marcel Mauss* (Lévi-Strauss, 1987 [1950]). In this succinct analysis of the latter's body of work, Lévi-Strauss addresses the question of the subjective implication of the anthropologist in his observations of other societies, which we consider to be a specific formulation of the question with regard to the relation between the individual and the collective. Mauss' concept of the total man implies that the anthropologist, as an observer, is of the same nature as his object of observation. Not only does he approach this society as an object of study, he also participates as a subject in this so-called object. Moreover, he has to externalize his subjective experience in order to present it in a formalized manner. This task would be impossible if the subjective and the objective would not meet at a given point.

Lévi-Strauss is prompted to formulate an answer to this question by an incongruity he perceives in Mauss' essay on the gift (Mauss, 2011 [1925]). In this seminal work, Mauss describes the principles of the exchange of gifts in different cultures. The study yields several observed principles, which can be found among very differing cultures across the globe: the obligation to give, to receive and to reciprocate. Moreover, many cultures formulate an explanation for these principles in terms of their own systems of belief. For example, the Maori of New Zealand refer to the *hau*. This is a spiritual essence which follows the gift wherever it goes and has the tendency to return to its origins. Therefore, if A offers a gift to B, and B passes this gift on to C, then C has to reciprocate to A for the *hau* present in the gift needs to be returned to its original source. However, Lévi-Strauss points out that we should never take these indigenous explanations at face value. A concept such as the *hau* is an element within a given symbolic system governing the exchange of gifts and as such should be scrutinized as to its function within this system.

He compares the *hau* and similar concepts with algebraic notions, which represent an undefined value of meaning but are themselves completely devoid of meaning. Their function is to reconcile the gap between signifier and signified. They are an attempt to restore a previously lost unity. As such, they do not represent an affective value, as Mauss arguments, but have a logical function and are to be situated on the same level as the relation they attempt to construct, which is a symbolic level. Whether something, an object, belongs to one person or the other is but a derivative of the original, relational character of the underlying reality. Indeed, according to Lévi-Strauss and in line with Mauss' own holistic approach of the total man, the underlying reality of these obligations and their uniting principle of the *hau* is the *totality of the exchange*. This totality is reflected in certain linguistic expressions used by some primitive cultures where giving and receiving, or borrowing and lending, are designated by one and the same word. As in any form of communication, the different terms implied (i.e., sender, receiver, gift, or message) are but secondary elements which divide an underlying, unified reality.

Lévi-Strauss stresses the relational character of the symbolic. Through the use of discrete elements it attempts to differentiate this previously undifferentiated reality. Terms such as subject and object, individual and collective, I and other, are differentiated through the use of the symbolic system. This is especially palpable in the principles present in the exchange of gifts. However, in such a system there is hardly any place for an individual separate from the collective. Indeed, Lévi-Strauss stresses the fact that in Mauss' study of the notion of the person (Mauss, 1938) the individual is relegated to a logical function within the symbolic system—which can only be collective.

## Lacan: the logic of the collective and the assertion of the subject

We have already discussed Lacan's approach of the relation to the other in terms of identification, which is ubiquitous in his paper on the family complexes. However, the events of World War II have brought the dire effects of identification to Lacan's attention (Roudinesco, 1997 [1993]). Moreover, a visit to England in 1947 acquainted him with the approach of Rickman and Bion in working with groups of mental patients during the war. Their views of group therapy were based on a conceptualization of the group in terms of horizontal identification (Bion and Rickman, 1943)^2^.

In 1953 Lacan wrote a letter to his ex-analyst, Rudolph Loewenstein, where he claims he has reached a point where he feels himself “a man more certain of his duties and his destiny” who has a growing assurance of what he has to say on “an experience of which only the last few years have permitted [him] to recognize the nature” (Lacan, 1976 [1953a], p. 132, our translation). This also marks the year of his *Rome Discourse* (Lacan, 2006 [1953b]), a text in which Lévi-Strauss' influence can all but be denied. However, in the years preceding it we see Lacan as a seeker, attempting not only to conceptualize a relation between the subject and the other that does not merely rely on identification, but also to formalize this relation in a logical system. Traces of his quest can be found in his papers on the logic of the collective (Lacan, 2001 [1945–1946], 2006 [1945]).

In a rare and rather dense paper Lacan (2001 [1945–1946]) presents us with a mathematical riddle which reveals the principle of a logical connection between a group and the individuals that constitute it. We are offered 12 visually identical coins, amongst which one can be discerned on grounds of its weight. We do not know whether this coin, the “bad coin,” is lighter or heavier than the others. Its quality does not concern us, the only thing that concerns us is the notion of its absolute difference. Furthermore, we have at our disposal a classic pair of scales. Our task is to discern the “bad coin” with a maximum of three separate weighings. We lack the space to expound the complete solution to this riddle (an excellent translation of Lacan's article can be found on-line). We will content ourselves with a brief summary of Lacan's proceedings and his conclusions.

After he has illustrated the solution to the problem as it is presented, Lacan ups the ante by adding another coin to the collection. Can we still solve the riddle if we have to discern the “bad coin” among 13 coins? This can indeed be done, provided that we use another procedure than the one applied to a collection of 12 pieces. Moreover, this new procedure permits Lacan to elaborate a formula that allows one to find the “bad piece” among a maximum amount of coins with the aid of a minimum amount of weighings. One merely has to follow three simple principles: first, one has to isolate a piece that is free of any suspicion; next, one brings about a discord in the distribution of suspicion (e.g., the amount of coins that is suspected to be lighter than the others is greater than the amount that is suspected to be heavier); finally, one discriminates between the remaining pieces until one has found the “bad piece.”

Without the complete solution at one's disposal this will undoubtedly sound complicated—and it is. However, the important thing to retain is the fact that this collection cannot be defined with the aid of any external criterium. There is no unifying characteristic other than the uniformity of the collection. The only way we can discern the “bad coin” is through a comparison with the other coins. Even when, in the first step, we isolate a coin that serves as a norm with which to compare the other coins, this is done through a comparison with the coins already present. In other words, the absolute difference that constitutes the individual in this collective can only be reached through a comparison with the others. Moreover, this relation between the individual and the collective can be formalized through a logical formula. As such, Lacan can sustain the notion of a separation between the individual and the collective (which was too radical in Durkheim's case) while at the same time formalizing this relation as a logical function (which was Lévi-Strauss' aim). In a paper that chronologically precedes this one, but which logically forms its consequence, Lacan (2006 [1945]) formulates how the subject asserts itself against the collective.

Lacan presents us with yet another riddle. Three prisoners are summoned by the warden who promises to grant one his freedom if he successfully stands a test. He presents them with five disks: three white and two black. Each prisoner will have a disc attached to his back such that he himself cannot see the color of his own disc, but can perfectly observe the disks the other two are wearing. Moreover, they are not allowed to communicate. The purpose of the test is to infer what color disc one is wearing based on logical reason. Thereupon, the warden distributes the white disks among the prisoners. After staring at each other for some time, all three prisoners head for the warden. What happened^3^.

In the ideal solution one prisoner, A, starts from the hypothesis that he is black. Within this condition another prisoner, B, could make the same hypothesis and easily come to the conclusion. If he were indeed black, the third prisoner, C, would see two black disks and leave at once. However, C does not move at once. Therefore, B can conclude that he is not black and leave. Yet, B does not leave either. Thus, A can only conclude that his initial hypothesis (“I am black”) was false. And because in reality all three prisoners followed the same path of reasoning, they all leave once they have reached this conclusion.

Lacan is hard pressed to point out the sophistic nature of this solution. For if they all leave at the same time, they all must doubt their initial conclusion which was based on the fact that the others remained standing still. Therefore, after they have all left, their doubts will make them all pause. This scenario is reminiscent of Achilles and the tortoise, and one can wonder whether they will ever reach the warden. Lacan arguments that they will, and that they only need two stops to come to an absolute, logical certainty. For the first halt objectifies B's conclusion: if C had seen two black disks, he would never pause. The second stop objectifies A's conclusion: if B pauses a second time, that means the first pause was not conclusive for him (which it would have been had A been black). Thus, Lacan concludes, these temporal hesitations are a necessity in order to arrive at a logically sound argument.

The introduction of time in order to arrive at a logical conclusion does not agree with the spatial nature of classical logic, which is based on the universality of certain forms. One does not need time: either one sees the solution or one does not see it. However, Lacan states that in this logic of the collective three logical times can be discerned. The first is what he call the *instant of the glance*. The riddle would be limited to this time if two black disks were distributed, its solution summarized in the following statement: “Being opposite two blacks, one knows that one is white,” (Lacan, 2006 [1945], p. 167). The subject of this statement is the impersonal “one” of every logical statement. This is also the subject present in Durkheim's mechanical solidarity: the “one” that represents the collective consciousness and is expressed through penal law. Indeed, according to Durkheim the latter does not imply that “I do not perform a certain act because it is punishable,” but rather that “it is punishable, because one does not perform such acts.” However, this subject is also present in the reasoning of Mauss and Lévi-Strauss as it can be compared to notions such as the *hau*. It merely serves a logical function within a closed, symbolic system. Yet Lacan has something different in mind, namely a subject that asserts itself against the collective. In order to arrive at this assertion, the subject has to encounter the other.

Indeed, the situation where one prisoner sees two black disks does not present itself. Therefore, a second time is inaugurated with the following (intuitive) statement: “Were I a black, the two whites that I see would waste no time realizing that they are whites,” (Lacan, 2006 [1945], p. 168). In this time, one prisoner (A in our example) makes himself the object of the gaze of the other two and puts himself in their position. In this case, time is necessary for the other two to reach a conclusion because it is based on the fact that the other stands still (which is interpreted as a hesitation). Lacan calls this the *time for comprehending*. The subject of this time is a subject determined by the reciprocity of the other. The relation between these subject is of an imaginary nature, meaning that the prisoners mirror each other (Aucremane, 1985).

The time for comprehending also puts every subject under a logical pressure. Indeed, how long does this comprehending take? If A hesitates to come to a conclusion and the other two precede him, he will never *ever* be able to reach any sound conclusion because it can only be based on their standing still. Therefore, a necessary *moment of concluding* interrupts the time for comprehending through the following statement: “I hasten to declare myself a white, so that these whites, whom I consider in this way, do not precede me in recognizing themselves for what they are,” (Lacan, 2006 [1945], p. 168). This statement is the subjective assertion. The time for comprehending leads to a conclusion, but only if the subject anticipates the certainty of his conclusion and seizes it in a moment of urgency.

Lacan operates a shift from spatiality to temporality, from a subject seized by an intersubjective logic to a subject that asserts itself in an act based on a judgment that lacks sufficient logical ground. This subject is the personal subject, the one that pronounces “I” (in French: “je”). It can only exist on the condition that it has assumed the other forms of subjectivity (i.e., the impersonal “one” and the reciprocal subject). The assertion of this subject is different from the subject as a mere function of the symbolic system—*and yet*, Lacan states that this subject also has a logical form and stems from a logical process.

According to Lacan the temporal logic of this riddle can be applied to group of undefined size, given that there is always one black disc less than there are prisoners. As in the case with the 13 coins, the group is not constituted by a common trait. The trait is retroactively defined by the group through the assertion of the subject. The collective is founded on the reciprocity of the difference. As such, the collective logic can be expressed according to the following principles: “(1) A man knows what is not a man; (2) Men recognize themselves among themselves as men; (3) I declare myself to be a man for fear of being convinced by men that I am not a man,” (Lacan, 2006 [1945], p. 174). Thus Lacan formalizes the relation between the individual and the collective. In this formalization the individual is a subject and as such a function within a logical process. However, through the anticipation of its truth it can assert itself against this collective and escape the reciprocal relation in which it was captured.

## Conclusion

Lacan's views on the relation between the subject and the other gradually change between 1938 and 1953. Initially this relation is conceived of as strictly based on the principle of identification. Such was the case in his paper on the family complexes, where the process of socialization is described as a series of consecutive identifications. From 1953 onward Lacan conceptualizes the Other no longer as an image with which to identify, but as a symbolic system governed by the laws of language. This new conceptualization is realized through the tools handed to him by Lévi-Strauss' structural anthropology. In this paper we have demonstrated that between 1938 and 1953 Lacan attempted to formulate the individual as a function, the subject, within a logic of the collective. Thus, although Lévi-Strauss handed him the methodology, this encounter does not constitute a radical breach in Lacan's work—it is merely a refining of the work he had already started.

Moreover, we have demonstrated that Lacan's statements on the logic of the collective can be situated within a broader frame of reference. Whereas Durkheim ended in a situation where individual and collective are radically separated, Mauss and Lévi-Strauss relegated the individual to a mere logical function within a collective, symbolic system. Lacan on the other hand conceptualized a subject that can assert itself against the other while still remaining part of a logical system. This is the conundrum that Lacan will confront time and again throughout his teachings and needs to be taken into account when one wishes to study the further influence of Lévi-Strauss on Lacan. The subject, a logical function within the Symbolic, speaks and asserts itself.

## Author contributions

The author confirms being the sole contributor of this work and approved it for publication.

### Conflict of interest statement

The author declares that the research was conducted in the absence of any commercial or financial relationships that could be construed as a potential conflict of interest.

## Footnotes

^1^Lévi-Strauss (1963 [1949]), in his paper on “The Effectiveness of Symbols,” gives an interesting pendant of this phenomenon. He explains how a shaman cures a woman experiencing difficulties during labor by connecting her symptoms with the collectively shared mythology, which has an effect on these physical symptoms. This paper has heavily influenced Lacan in his interpretation of the Symbolic Order (Zafiropulos, 2010 [2003]).

^2^This contrasts with the Freudian point of view, where the vertical identification with the leader takes precedence over the horizontal identifications between the members of the group (Freud, 1955 [1921]).

^3^We will only scratch the surface of this text. For a thorough discussion of the riddle, its solution and implications, see Hoens (in press).
